# Supplementary material for: Hydrogen Gas Sensing Properties of Mixed Copper–Titanium Oxide Thin Films
Source: Sensors (Basel). 2023 Apr 8;23(8):3822. doi: 10.3390/s23083822 (PMC10144612; doi:10.3390/s23083822)
Supplement: Supplementary file 1 [file sensors-23-03822-s001.zip › sensors-2234552-supplementary.pdf]

Supplementary Materials

Hydrogen gas-sensing properties of mixed copper–titanium oxide thin films

Ewa Mańkowska [1], Michał Mazur [1*], Jarosław Domaradzki [1], Piotr Mazur [2], Małgorzata Kot [3] and Jan Ingo Flege [3]

[1] Faculty of Electronic, Photonics and Microsystems, Wrocław University of Science and Technology, Janiszewskiego 11/17, 50-372 Wrocław (Poland)
[2] Institute of Experimental Physics, University of Wrocław, Max Born 9, 50-204 Wroclaw (Poland)
[3] Applied Physics and Semiconductor Spectroscopy, Brandenburg University of Technology Cottbus-Senftenberg, Konrad-Zuse-Strasse 1, Cottbus (Germany)
*Correspondence: michal.mazur@pwr.edu.pl

XPS measurements were performed to determine the surface chemistry of the prepared oxides before and after annealing. As can be seen in Figure S1, before the annealing process, in addition to components related to Cu, Ti, and O, relatively high concentrations of carbon were found on all samples surfaces. This is most probably due to their long storage time at ambient conditions prior to the measurements. In addition to carbon, we found a signal from nitrogen in the samples with 23 and 56 at.% Cu, which may originate from the residue of the Kapton tape that was used on the sample before XPS characterization.

Titanium was only found on the sample surface with 77 at.% of Cu. The reason for this observation could be that this sample had the lowest amount of carbon contamination on its surface, thereby facilitating titanium detection by the very surface-sensitive XPS method. It should be noted that the prepared mixed oxide samples have titanium in their composition, as shown in [9]. After annealing all samples in the furnace at 473 K at the same time, the majority of the carbon, nitrogen, and titanium signal was removed for all samples surfaces. We believe that after the annealing process the titanium is covered by copper, as it is known that copper can migrate towards the surface during annealing at elevated temperatures. In addition, we detected a very small signal from copper (i.e., less than 1.5% of all components visible in the spectrum) in the XPS spectra of the TiO_x sample. Therefore, we suppose that a very small amount of copper cross-contaminated the TiO_x sample surface during the annealing process in the same tube in a furnace. Furthermore, in the XPS spectra after annealing we detected signals at the binding energy positions of SiO_2 in the Si2p and Si2s core levels. With all of this knowledge, we tried to fit our detailed XPS spectra of the O1s core level.

The O1s spectra of the as-prepared samples were decomposed into several peaks (if applicable) related to Ti (530.1 eV) and Cu (II) (529.9 eV) oxides [50,51], defective oxides or metal carbonates (531.4 eV), and adsorbed –OH groups or organic carbon (532.7 eV) (Figure S2). Before annealing, in most cases the O1s signal is dominated by the peak at 531.4 eV, which, depending on the amount of adsorbed contaminants, is then followed by the peaks related to the presence of Ti and/or Cu oxides. Additionally, adsorbed –OH groups or organic carbon (C-O) were found on all sample surfaces, mostly on the TiO_x sample. As non-negligible N1s peak intensities were observed for the as-prepared samples with 23 and 56 at.% of Cu, potential formation of oxynitrides (O-N) was considered [1,2].

After annealing, the major peak was from the metal oxide signals (copper and titanium oxides), although a few adsorbed contaminants or defects (peaks at around 531.4 eV) were found on these oxides' surfaces. In addition, as mentioned above, we found a signal from the SiO_2 substrate after annealing that was located around 532.7 eV in the O1s XPS, where –OH or organic carbon species appear as well. However, we believe that this signal after annealing is now more related to the SiO_2 than to the other contaminants, as its intensity trend appears to be correlated with the trends in Si 2p and Si 2s intensity that we found in the overview spectra for these samples. Indeed, we found the highest Si signal

in the XPS results of the sample with 56 at.% of Cu in the mixed oxide film, and the peak at 532.7 eV is the highest O1s component in this sample. Most likely the Si signal in the XPS spectra originates from areas where the sample was held during the magnetron sputtering process due to the XPS spot being integrated over this region; alternatively, annealing may have induced local opening or breaking of the film that resulted in the evolution of micropores, thereby allowing partial probing of the substrate.

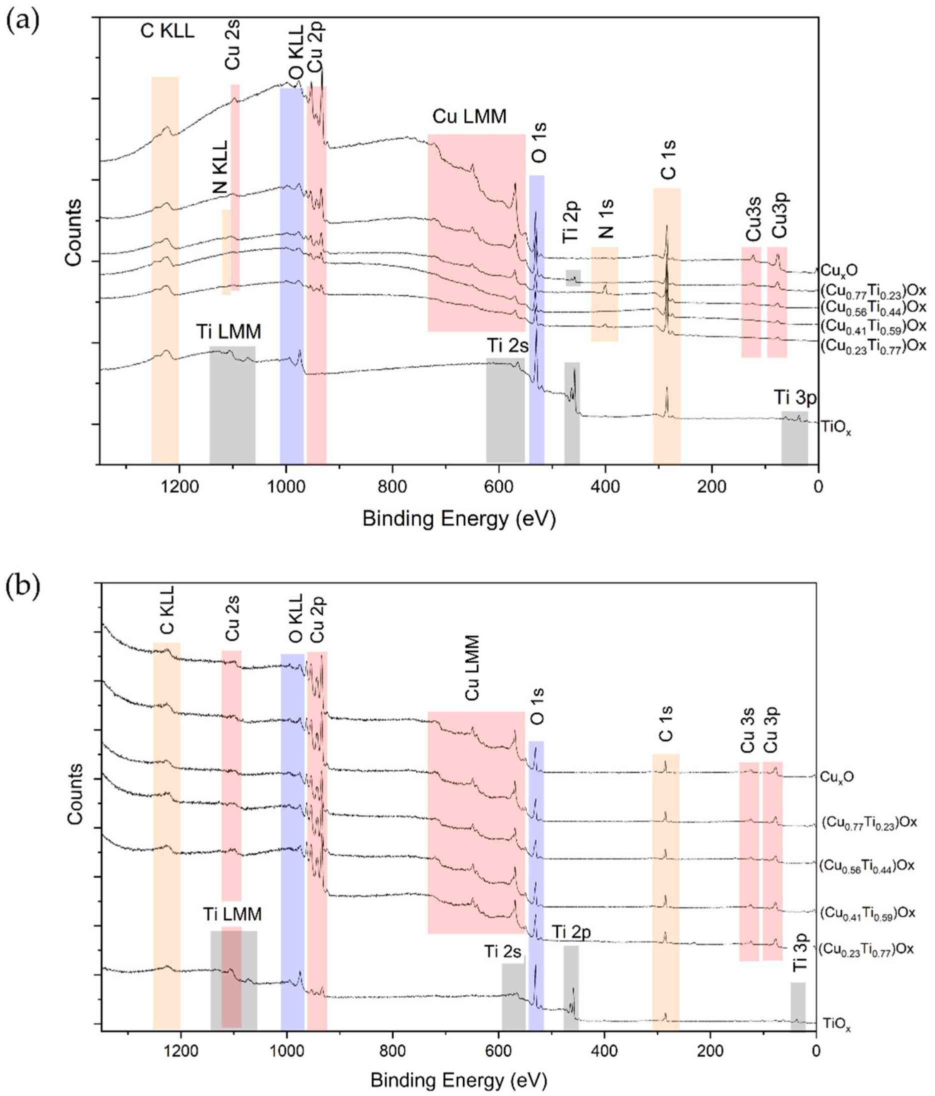

Figure S1. XPS survey spectra of the as-deposited (a) and annealed at 473 K (b) TiO_x, (CuTi)Ox, and Cu_xO thin films.

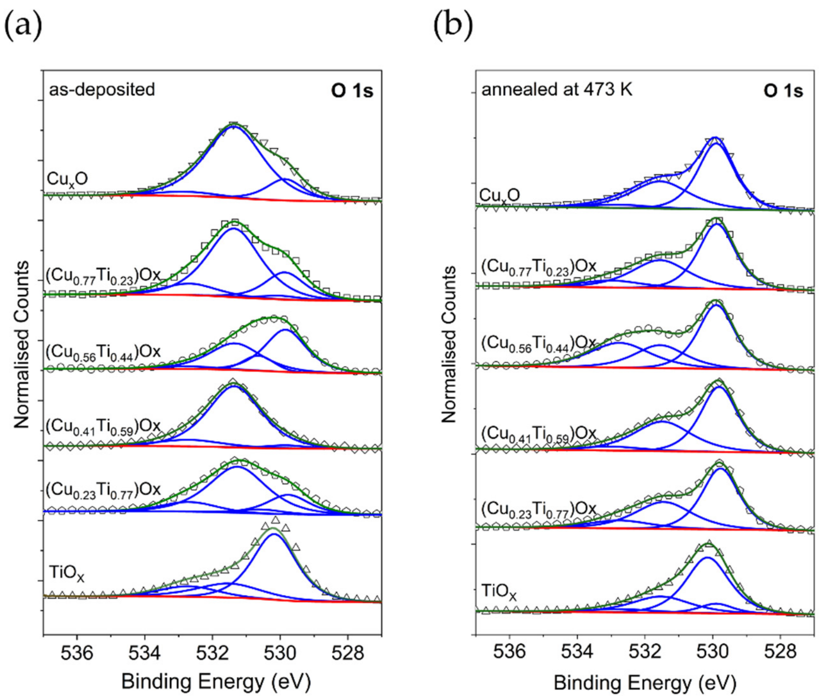

Figure S2. The O1s core level XPS spectra of TiOx, (CuTi)Ox, and CuxO thin films: (a) after deposition and (b) after annealing at 473 K.

References

[1] Ruiz-Cañas, M.C.; Quintero, H.I.; Corredor, L.M.; Manrique, E.; Romero Bohórquez, A.R. New Nanohybrid Based on Hydrolyzed Polyacrylamide and Silica Nanoparticles: Morphological, Structural and Thermal Properties. *Polymers* **2020**, *12*, 1152. https://doi.org/10.3390/polym12051152

[2] Ayiania, M., Smith, M., Hensley, A.J.R., Scudiero, L., McEwen, J.-S. and Garcia-Perez, M. Deconvoluting the XPS spectra for nitrogen-doped chars: An analysis from first principles. *Carbon* **2020**, *162*, 528. https://doi.org/10.1016/j.carbon.2020.02.065
